# Reviewer acknowledgement 2013

**DOI:** 10.1186/1471-2318-14-11

**Published:** 2014-03-18

**Authors:** Irene Pala

**Affiliations:** 1 BioMed Central, 236 Gray’s Inn, Road, London WC1X 8HB, UK

## Abstract

**Contributing reviewers:**

The editors of BMC Geriatrics would like to thank all our reviewers who have contributed their time to the journal in Volume 13 (2013).

## 

Mona Kristin Aaslund

Norway

Ivo Abraham

USA

Kathleen Abrahamson

USA

Wilco Achterberg

Netherlands

Daisy Acosta

Dominican Republic

Kathryn Adams

USA

Geri Adler

USA

Kristina Akesson

Sweden

Stephen Alway

USA

Bruno Amato

Italy

Melissa K Andrew

Canada

Mahesan Anpalahan

Australia

Ildebrando Appollonio

Italy

Torunn Askim

Norway

Jose Alberto Avila-Funes

Mexico

Majda Azermai

Belgium

Gillian Bartlett

Canada

Vivianne Baur

Netherlands

Clemens Becker

Germany

Christian Benedict

Sweden

Petra Benzinger

Germany

Sarah Berdot

France

Astrid Bergland

Norway

Angelo Bianchetti

Italy

Claudio Bilotta

Italy

Wendy Birmingham

USA

Hubert Blain

France

Alessandro Ble

Italy

Michel Bleijlevens

Netherlands

Jeanet Blom

Netherlands

Klaus Boes

Germany

Bienvenu Bongue

France

Willem Bossers

Netherlands

Kim Bouillon

UK

Marguerite Bramble

Australia

Barbara Brandt

USA

Sandra Brauer

Australia

Gina Bravo

Canada

Gisela Büchele

Germany

Allison Burfield

USA

Bianca Maria Buurman

Netherlands

Gerard Byrne

Australia

Francesco Cacciatore

Italy

Priscila Caçola

USA

Ian Cameron

Australia

David Cameron-Smith

New Zealand

Gideon Caplan

Australia

Julie Cartwright

USA

Robin Casten

USA

Sing Mei Chan

Canada

Liang-Chih Chang

Taiwan

Huashuai Chen

USA

Jian Sheng Chen

Australia

Lei Chen

USA

Karen Siu Lan Cheung

Hong Kong

Iris Chi

USA

Jill Chonody

USA

Lene Christiansen

Denmark

Andrew Clegg

UK

Remy Coeytaux

USA

Mauro Colombo

Italy

Giovanni Corso

Italy

Helen Creases

Australia

Teresa Damush

USA

Eling D de Bruin

Switzerland

Jan De Lepeleire

Belgium

Anke de Veer

Netherlands

Nienke de Vries

Netherlands

Anja Declercq

Belgium

Eric Decloedt

South Africa

Matthew Delmonico

USA

Wendy den Elzen

Netherlands

Michael Denkinger

Germany

Joseph Devaney

USA

John Devlin

USA

Anne Dewar

Canada

Marija Djukic

Germany

Huan-Ji Dong

Sweden

Rose-Marie Droes

Netherlands

Wendy Duggleby

Canada

Gordon Duncan

UK

Gammon Earhart

USA

Charlotte Edwardson

UK

Renske Eilers

Netherlands

Ann Catrine Eldh

Sweden

Nina Emaus

Norway

Coralie English

Australia

Mariani Erminia

Italy

Mary Ersek

USA

William Evans

USA

Mark Fagan

Norway

Qiushi Feng

Singapore

Assumpta Ferrer

Spain

Lois Finch

Canada

Kathleen Finlayson

Australia

Johan Flamaing

Belgium

Steffen Fleischer

Germany

Jane Fleming

UK

Marc Fleming

USA

Dorothy Forbes

Canada

Chris Fox

UK

Ellen Freiberger

Germany

Jong-Ling Fuh

Taiwan

Terry Fulmer

USA

Catharine Gale

UK

Gaurav Garg

Sweden

Hilde Geraedts

Netherlands

William Gibson

UK

Helen Gilburt

UK

Adrienne Gilligan

USA

Andrea Giusti

Italy

Robbert Gobbens

Netherlands

Stefan Goemaere

Belgium

Claire Goodman

UK

Bamini Gopinath

Australia

Elmar Graessel

Germany

James Graham

USA

Ann Gruber-Baldini

USA

Maria Grypdonck

Belgium

Yves Gschwind

Australia

Danan Gu

USA

Antonio Guaita

Italy

Valeri Guajardo

Brazil

Jason Guertin

Canada

Jun Guo

China

Narcis Gusi

Spain

Mark Haddad

UK

Andreana Haley

USA

Stephen Halloran

UK

Laura Hanson

USA

Nigel Harris

UK

Christine Hartmann

USA

Jorunn Helbostad

Norway

Manuel Hernandez

USA

Edson Hirata

Brazil

Marcus Hollander

Canada

Dirk Houttekier

Belgium

Ruth Hubbard

Australia

William Hung

USA

Paulette Hunter

Canada

Krista Huybrechts

USA

Andrea Icks

Germany

Shinya Ishii

Japan

Carlos Jaramillo

USA

Shannon Jarrott

USA

Herbert Jelinek

Australia

Sharon Kaasalainen

Canada

Sundaran Kada

Norway

Ichiro Kai

Japan

Masahiro Kamouchi

Japan

hulagu kaptan

Turkey

Saija Karinkanta

Finland

Gertrudis IJM Kempen

Netherlands

Dave Kerby

USA

Marie-Jeanne Kergoat

Canada

Irena Keser

Croatia

Sunil Khanna

USA

Hunkyung Kim

Japan

Hye-Kyung Kim

South Korea

Janice Knoefel

USA

Susan Helen Koch

Australia

Helen Kohlen

Germany

Kono Koichi

Japan

Sascha Köpke

Germany

Nienke Kosse

Netherlands

Alexander Kulminski

USA

Mehmet Kuyumcu

Turkey

Daniel Lai

Canada

Linda Lam

Hong Kong

Tzuo-Yun Lan

Taiwan

Laura Laslett

Australia

David Le Couteur

Australia

Wei-Ju Lee

Taiwan

Nancy Lerner

USA

Gill Lewin

Australia

Melanie Logue

USA

Vivian W Q Lou

Hong Kong

Lee-Fay Low

Australia

Robyn Lucas

Australia

Terry Lum

Hong Kong

Weixiang Luo

Hong Kong

Ken Madden

Canada

Jane Mahoney

USA

Daniel Malone

USA

Richard Marottoli

USA

Elisa Marques

Portugal

Pilar Martinez

Netherlands

Shino Matsuura

Japan

Kathleen McCauley

USA

Colleen McHorney

USA

Harry McNaughton

New Zealand

René JF Melis

Netherlands

Jasmine Menant

Australia

Graydon Meneilly

Canada

Janet Mentes

USA

Thomas Meuser

USA

Gabriele Meyer

Germany

José C Millán-Calenti

Spain

Martha Mohler

USA

Ralph Möhler

Germany

Sara Mondini

Italy

S Monfardini

Italy

Ali Montazeri

Iran

Kirsten Moore

Australia

Spencer Moore

Canada

Alessandro Morandi

Italy

Sara Morghen

Italy

Mario Luca Morieri

Italy

Vikky Morris

UK

Claire Morrisby

Australia

Donald Morrish

Canada

Enrico Mossello

Italy

Gabriele Müller-Mundt

Germany

Kathy Murphy

Ireland

Geoff Murray

Australia

Maria Nabal

Spain

Satoko Nagata

Japan

Miharu Nakanishi

Japan

Malavolta Nazzarena

Italy

Rick Nelson

UK

Rick Nelson

USA

Karin Neufeld

USA

Alessandro Nobili

Italy

Yu Nofuji

Japan

Louise Nygård

Sweden

Yoshiro Okubo

Japan

Marcel Olde Rikkert

Netherlands

Tone Kristin Omsland

Norway

Graziano Onder

Italy

Hannah O'Rourke

Canada

Peter Oster

Germany

Roberto Paganelli

Italy

Elena Paillaud

France

Pradeep Pallati

USA

Jorma Panula

Finland

Heekyeong Park

USA

Luca Pasina

Italy

Nancye May Peel

Australia

Geeske Peeters

Australia

Julie Peltier

France

Sandra Pereira

Portugal

Subashan Perera

USA

Chetan Phadke

Canada

Elizabeth Phelan

USA

Anastas Philalithis

Greece

Arwen Pieterse

Netherlands

Giulio Pioli

Italy

Francesco Pizzarelli

Italy

Jenny Ploeg

Canada

Graham Pluck

Japan

Michele Poletti

Italy

Jeff Poss

Canada

Mary Jo Pugh

USA

Martine Puts

Canada

Katayoun Rabiei

Iran

Anette Hylen Ranhoff

Norway

Kilian Rapp

Germany

Michael Rapp

Germany

Irene Maeve Rea

UK

Elizabeth Regan

USA

Borje Rehn

Sweden

Matthias Reinhard

Germany

Anna Renom-Guiteras

Germany

Sandra Reynolds

USA

Lothar Rink

Germany

Craig Ritchie

UK

Leocadio Rodríguez-Mañas

Spain

Martin Roland

UK

Elisabeth Romme

Netherlands

Bjorn Rosengren

Sweden

David Russell

USA

Marc Sabbe

Belgium

Catherine Said

Australia

Ritva Sakari

Finland

Ingvild Saltvedt

Norway

Fabio Salvi

Italy

Elizabeth Sampson

UK

Jessica Sautter

USA

Hélène Sauzéon

France

Ingmar Schafer

Germany

Marieke Schuurmans

Netherlands

Rene Schwendimann

Switzerland

Michael Schwenk

USA

David Scott

Australia

Satoshi Seino

Japan

Dallas Seitz

Canada

Geir Selbak

Norway

Davide Seripa

Italy

Lisa Shaw

UK

Aubrey Sheiham

UK

Paul Shelton

USA

Hyehyung Shin

South Korea

Jin Hee Shin

USA

Olayinka Shiyanbola

USA

Doungkamol Sindhusake

Australia

Cody Sipe

USA

Sarianna Sipilä

Finland

Zbigniew Siudak

Poland

Martin Smalbrugge

Netherlands

Alex Smithson

Spain

Roy L Soiza

UK

Yuki Soma

Japan

Silvia Sorensen

USA

Vincent Staggs

USA

Els Steeman

Belgium

Richard Stefanacci

USA

Eileen Stock

USA

Carsten Strobel

Norway

Daina Sturnieks

Australia

Eileen Sullivan-Marx

USA

Esther Suter

Canada

Christian Swine

Belgium

Sarah Szanton

USA

Pawel Szulc

France

Erwin Tak

Netherlands

Hideto Takahashi

Japan

Kristine Talley

USA

Nanako Tamiya

Japan

Der-Cherng Tarng

Taiwan

Charuhas Thakar

USA

Olga Theou

Canada

Stephen Thielke

USA

Genevieve Thompson

Canada

Christophe Trivalle

France

Hung-Bin Tsai

Taiwan

Pao-Feng Tsai

USA

Mimi Tse

Hong Kong

Kenji Tsunoda

Japan

Lieve Van den Block

Belgium

Hendrik van den Bussche

Germany

Jenny Theodora van der Steen

Netherlands

Nathalie van der Velde

Netherlands

Marie-Claire Van Nes

Belgium

Peter van Vliet

Netherlands

Jessie VanSwearingen

USA

Massimo Venturelli

Italy

Hilde Verbeek

Netherlands

Anne G Vinsnes

Norway

Pieter Jelle Visser

Netherlands

Trang Vu

Australia

Åke Wahlin

Sweden

Jonathan Waite

UK

Wei Wang

Australia

Shuichiro Watanabe

Japan

Paul Welsh

UK

Joseph Wherton

UK

Darryl Wieland

USA

Jenny Wilkinson

Australia

Jacek M Witkowski

Poland

Karin Wolf-Ostermann

Germany

Martin Wolkewitz

Germany

Bettina Wollesen

Germany

Jean Woo

Hong Kong

Michael Woodward

Australia

Yijian Yang

Canada

Yiqing Yang

USA

Nobufumi Yasuda

Japan

Andrea Yevchak

USA

Abebaw Mengistu Yohannes

UK

Lukas Zahner

Switzerland

Astrid Zech

Germany

Lei Zhang

China

Li Zhang

China

Junshan Zhou

China

Peter Zock

Netherlands

Gilbert Zulian

Switzerland

Giovanni Zuliani

Italy

Sandra Zwakhalen

Netherlands

## Pre-publication history

The pre-publication history for this paper can be accessed here:

http://www.biomedcentral.com/1471-2318/14/11/prepub

